# Constant–Current Coulometry with Electrogenerated Titrants as a Novel Tool for the Essential Oils Screening Using Total Antioxidant Parameters

**DOI:** 10.3390/antiox11091749

**Published:** 2022-09-03

**Authors:** Guzel Ziyatdinova, Alena Kalmykova, Olga Kupriyanova

**Affiliations:** 1 Analytical Chemistry Department, Kazan Federal University, Kremleyevskaya 18, 420008 Kazan, Russia; 2 Institute of Fundamental Medicine and Biology, Kazan Federal University, Kremleyevskaya 18, 420008 Kazan, Russia; 3 Regional Research and Testing Center “Pharmexpert”, Kazan State Medical University, Tolstogo 6/30, 420012 Kazan, Russia

**Keywords:** total antioxidant capacity, ferric reducing power, coulometry, electrogenerated titrants, antioxidants, essential oils

## Abstract

Essential oils are widely used in aromatherapy, medicine, and food industries due to a wide spectrum of bioactivity. Their antioxidant properties can be considered as markers of therapeutic effect and quality. Constant–current coulometry with electrogenerated titrants has been successfully applied for these purposes for the first time. Fifteen types of essential oils from various plant materials have been studied. Their composition has been identified by gas chromatography with mass-spectrometric detection (GC-MS). The reactivity of individual antioxidants of essential oils towards electrogenerated titrants (bromine and ferricyanide ions) has been estimated. Total antioxidant parameters, in particular total antioxidant capacity (TAC) and ferric reducing power (FRP) based on the reactions of essential oil antioxidants with electrogenerated bromine and ferricyanide ions, respectively, have been evaluated. Positive correlations (*r* = 0.7051–0.9558) with common antioxidant tests (antioxidant activity by reaction with 2,2-diphenyl-1-picrylhydrazyl (DPPH^•^) and total phenolic content by the Folin–Ciocalteu method) have been obtained. Coulometric approaches overcome the limitations of spectrophotometry and are applicable to a wider range of essential oils.

## 1. Introduction

Plant materials are widely studied objects in different branches of chemistry. One of the intensively developed directions in this field is the investigation of the antioxidant properties and their impact on human health. Various types of plant material-based samples (extracts, decoctions, infusions, tinctures, oleoresins and essential oils) are in focus to date. Among them, essential oils are of practical interest due to their wide spectrum of biological activity and application area. In nature, essential oils are involved in the plant defense system and signaling processes. For example, plant aroma compounds participate in the protection of plants from microorganisms, insects, and herbivores, in the attraction of the pollinating insects and fruit-distributing animals, in water regulation, and allelopathic interactions [1].

Since ancient times, essential oils have been used in aromatherapy [2]. Antibacterial, anti-inflammatory, antioxidant and antiviral properties of essential oils are successfully used in the auxiliary therapy of a wide range of human diseases [3]. On the other hand, these properties make possible application of the essential oils as additives in the food industry to increase the shelf life of final products [4,5].

Essential oils are highly concentrated extracts from flowers, leaves, stems, fruits, and roots obtained by hydrodistillation, steam or dry distillation, as well as mechanically (for citrus fruits) [1]. The major constituents of essential oils determining their aromas and bioactivity are saturated and unsaturated hydrocarbons, alcohols, aldehydes, esters and ethers, ketones, phenols, and terpenes [6,7]. Many of them possess antioxidant properties allowing the usage of total antioxidant parameters for the essential oils’ characterization.

Antioxidant properties of essential oils are usually evaluated by spectrophotometric approaches [8] characterized by simplicity and cost-efficiency. Typical antioxidant parameters estimated are the antioxidant activity by reaction with 2,2-diphenyl-1-picrylhydrazyl (DPPH^•^) [9,10,11,12,13] or peroxyl radicals obtained from 2,2′-azino-bis(3-ethylbenzothiazoline-6-sulfonic acid) (ABTS^+•^) [11,12,13,14]. The total phenolics assay performed by the Folin–Ciocalteu method [15,16], ferric reducing-antioxidant power (FRAP) and β-carotene bleaching assay are used more rarely [9,13,14]. Furthermore, extracts obtained from corresponding plant materials are often studied instead of essential oil. Most published works are focused on the essential oils from one type of plant material, while the screening studies are almost out of consideration.

Further development in the field could be focused on the development of electrochemical approaches for the evaluation of essential oils’ antioxidant parameters as far as reactions of antioxidants proceed with the electron transfer that partially mimicks their action in living systems. Moreover, a wide range of electrochemical methods has been developed for the estimation of total antioxidant parameters of plant materials and their extracts [17,18,19,20,21,22,23,24]. Electrooxidation of the plant antioxidants at the electrode surface under conditions of chronocoulometry [17] and voltammetric monitoring of the reactions with DPPH^•^ [18] and galvinoxyl [19] radicals immobilized at the electrode surface as well as homogeneous reactions with electrochemically generated superoxide anion radicals [22,23,24] that have been successfully realized for the plant extracts, medicinal plant tinctures, infusions and decoctions. Unfortunately, essential oils almost do not get attention as samples of interest in electroanalytical estimation of total antioxidant parameters nowadays. Just one voltammetric approach based on the reaction of antioxidants with superoxide anion radicals has been reported with an example of the *Mentha* species [24]. The method is based on the decrease of oxygen electroreduction currents corresponding to the formation of superoxide anion radicals in the presence of essential oils. Differential pulse voltammetry has been used for reaction monitoring.

Summarizing that mentioned above, development of simple, reliable and cost-effective electrochemical approaches for the evaluation of total antioxidant parameters of essential oils is of practical interest. Constant–current coulometry with electrogenerated titrants has been shown to be a powerful tool for the estimation of total antioxidant parameters of different types of foodstuffs, biological fluids, spice extracts, and phytopharmaceuticals [25]. Halogens (iodine, bromine, and chlorine) and hypohalogenite ions are used as coulometric titrants. Halogens are generated from halogenides in acidic medium, while hypohalogenites in basic medium [25,26]. Electrogenerated halogens differ by oxidative strength, which is increased in the order iodine < bromine < chlorine. Bromine acting as an oxidant of average strength is the most suitable for evaluating the antioxidant properties of various samples. Iodine is too weak an oxidant and does not react with many antioxidants [25]. On the contrary, chlorine is a very strong oxidant leading to deep transformation of the antioxidants as well as to the side reactions that is reflected in the irreproducible stoichiometric coefficients of the titration [27]. Hypohalogenite are used in organic analysis for the determination of sulfanilamide preparations, acid hydrazides and amides, amino acids, and sulfur-containing pharmaceuticals which are stable in basic media [26].

The application of a coulometric approach to the analysis of essential oils will expand its analytical capabilities.

The aim of the present study is to show applicability of constant–current coulometry with electrogenerated titrants for the essential oils analysis. This type of reagent is favorable vs. radicals or other typical reagents due to the following reasons:

- No need to prepare a standard solution of the reagents i.e., the standardization stage is excluded;
- Titration is an absolute that excludes usage of calibration plots (in fact, an electron acts as a titrant);
- No effect of sample dilution;
- Coverage of almost all types of antioxidants based on titrants’ reactivity;
- Ease of calculation and the possibility of using different standard antioxidants;
- High sensitivity, reliability, and reproducibility of the measurements;
- Possibility of automation for routine applications.

Coulometric titrants (electrogenerated bromine and ferricyanide ions) have been used as reactive species. Their reactions with individual antioxidants of essential oils have been studied. The data obtained allow evaluation of the total parameters of essential oils in particular total antioxidant capacity (TAC) and ferric reducing power (FRP). Fifteen types of essential oils from various plant materials have been investigated and their components identification has been performed by GC-MS. The correlation analysis of total antioxidant parameters with antioxidant activity towards DPPH^•^ and total phenolic contents has been carried out.

## 2. Materials and Methods

### 2.1. Reagents and Samples under Investigation

Thymol of 99.5% purity (Sigma, Steinheim, Germany), 98% carvacrol, 99% eugenol, 98% syringaldehyde (Aldrich, Steinheim, Germany), 99% vanillin and 97% phythol (Sigma-Aldrich, Steinheim, Germany), 99% benzyl alcohol, 96% limonene, 98% α-pinene, 98% β-pinene, 98% D-carvone, 75% camphene, as well as α-fenchene, myrcene, 99.5% L-menthol, and 97% L-borneol (Acros Organics, Geel, Belgium) were used as standard antioxidants. Their 10 mM stock solutions were prepared by dissolving an accurately weighed portion in 5.0 mL of ethanol (rectificate). Less concentrated solutions were obtained with an exact dilution prior to measurements. Other reagents were of chemical purity and used as received.

The commercially available essential oils of various plant materials (Table 1) of different trademarks were studied. Their ten-fold dilution with ethanol was used exactly before the measurements.

### 2.2. Constant–Current Coulometry

Coulometric measurements were carried out on the coulometric analyzer “Exper-006” (Econix-Expert, Moscow, Russia) supplied with the electrochemical cell consisting of four electrodes: a working electrode (bare platinum wire with 0.5 cm^2^ surface area), an auxiliary electrode (platinum wire separated from the anodic compartment of the cell with the semipermeable membrane), and two indicator electrodes (platinum needle electrodes) for the detection of the titration end point. A polarizing potential of 200 mV was applied between the indicator electrodes. The surface of the platinum electrodes was cleaned with nitric acid followed by rinsing with distilled water.

Constant–current generation of coulometric titrants was performed at the current density of 5 mA cm^−2^ providing their 100% current yield. 0.2 M KBr in 0.1 M sulfuric acid and 0.1 M K_4_Fe(CN)_6_ in 2 M sodium hydroxide were used as precursors and supporting electrolytes for the generation of bromine and ferricyanide ions, respectively.

Coulometric titration was performed in a 50 mL cell containing 20.0 mL of the precursor in the supporting electrolyte. The generating circuit was switched on until a 40 µA indicator current was achieved. Then, an aliquot portion (10–100 μL) of the antioxidant standard solution or 10-fold diluted essential oil was added to the cell, and the timer was started simultaneously. The titration end-point was detected by achieving the initial value of the indicator current. The timer was stopped, and the generating circuit was turned off. Faraday’s law of electrolysis was used for the calculation of the number of electrons participating in the reaction of individual antioxidants with titrants.

The total antioxidant parameters, in particular the total antioxidant capacity and ferric reducing power, were evaluated for the essential oils as the quantity of electricity spent for the titration of the sample and recalculated per 1 mL of the essential oil.

### 2.3. GC-MS Identification of Essential Oil Components

Essential oils were dissolved in diethyl ether in 1:10 (*v*/*v*) ratio [28] and analyzed by GC-MS using an Agilent Technologies 7890B GC System equipped with a HP-5MS capillary column ((5%-phenyl)-methylpolysiloxane phase, 30 m, 0.25 mm i.d. and 0.25 μm film thickness) and mass-selective detector Agilent Technologies 5977A MSD (Agilent Technologies, Santa Clara, CA, USA). In addition, 1 µL of the diluted sample was injected in the 1:50 split and splitless mode. Acetylation, methylation and silylation procedures were performed using standard approaches [29,30,31]. The samples obtained were measured in splitless mode. GC analysis was performed under the following conditions: injector and detector temperatures were set at 280 °C. The column temperature was initially 50 °C for 2 min, then gradually increased to 240 °C at 3 °C min^−1^, and finally increased to 280 °C at 20 °C min^−1^ and kept at 280 °C for 5 min. Helium with a flow rate of 1 mL min^−1^ was used as a carrier gas. An electron ionization system with an ionization energy of 70 eV was used. Mass spectra were recorded with total ion currents over *m*/*z* 20−550 Da. The components identification was performed by comparison of the retention times and retention indices with reported ones [32,33] as well as by matching the mass-spectra obtained with data from NIST 14 (National Institute of Standards and Technologies, Mass Spectra Libraries, Palmer, MA, USA) [34]. The NIST MS Search 2.2 software (National Institute of Standards and Technologies, Mass Spectra Libraries, Palmer, MA, USA) was used for these purposes.

### 2.4. Antioxidant Activity Assay

DPPH^•^ stable radical (Aldrich, Steinheim, Germany) was used as a reagent to evaluate the antioxidant activity of essential oils. The standard procedure was applied [35]. Briefly, 4 μL of 10-fold diluted ethanol essential oil were thoroughly mixed with 3 mL of 0.10 mM DPPH^•^ methanol solution and incubated in the dark for 20 min. Then, the absorption was read at 515 nm vs. methanol with the addition of 4 μL of the sample using spectrophotometer PE-5300 (NPO Ecros, Saint Petersburg, Russia). Control DPPH^•^ absorption was measured vs. methanol. Antioxidant activity of the essential oils was expressed as a relative decrease of the DPPH^•^ absorption due to the reaction with the antioxidants of the sample.

### 2.5. Total Phenolics Assay

The Folin–Ciocalteu colorimetric method [36] with some modifications was used for the total phenolics quantification. In addition, 0.5 mL of the sample (10,000-fold diluted clove and thyme essential oils and 1000-fold diluted cinnamon and nutmeg essential oils) or standard solution of eugenol (5.00, 9.98, 25.0, 50.1, 75.4 and 100.5 mg L^−1^) were placed in a 5.0 mL volumetric flask. Then, 2.5 mL of diluted Folin–Ciocalteu reagent (1:10 (*v*/*v*)) (Aldrich, Steinheim, Germany) were added and thoroughly mixed. After 4 min, 2.5 mL of 7.5% Na_2_CO_3_ solution were added, mixed and incubated for 1 h. The absorbance of the solution was measured at 765 nm in a 0.5 cm cuvette. The blank solution contained all the reagents excluding essential oil, which was replaced with 0.5 mL of ethanol. Total phenolic content was expressed in eugenol equivalents per 1 mL of essential oil.

### 2.6. Statistics and Correlation Analysis

Five parallel measurements were performed for constant–current coulometry (three replications for chromatography and spectrophotometry). A significance level of 5% was used for the statistical treatment of the experimental data. The results were presented as an average value and a coverage interval. Relative standard deviation (RSD) was used for random error characterization.

Correlation analysis was performed using OriginPro 8.1 software (OriginLab, Northampton, MA, USA).

## 3. Results and Discussion

### 3.1. Essential Oils Characterization by GC-MS

The essential oils under consideration have been studied by GC-MS for the components identification. The number of constituents varied in a range of 30–79 depending on the type of essential oil (Table 2).

The major components of the essential oils are terpenes and their derivatives as well as phenolic volatile compounds (eugenol, carvacrol, thymol and methoxyphenolics). The most widely distributed terpenes are α-pinene, camphene, β-pinene, myrcene, tricyclene, α-thujene, *m*-cymene, limonene, eucalyptol, *trans*-β-ocimene, γ-terpinene, linalool, α-terpineol, linalyl acetate, α-copaene, β-elemene, *trans*-β-caryophyllene, caryophyllene oxide I, α-humulene, α- and γ-muurolene. Other terpenoids are more specific to the type of essential oil. The chromatographic data obtained agree well with those reported in [1,37,38,39] for essential oils of various plants.

Such composition of essential oils suggests the possibility of using constant–current coulometry with electrogenerated titrants to assess their antioxidant properties. Coulometric titration of essential oils antioxidants has been performed for confirmation.

### 3.2. Reactions of Individual Antioxidants with Electrogenerated Titrants

The reactivity of individual antioxidants of essential oils toward coulometric titrants (electrogenerated bromine and ferricyanide ions) has been studied. These two reagents are very different in properties.

Oxidation of bromide ions at a platinum electrode in acidic medium results in the formation of tribromide anion, molecular bromine and short-living bromine radicals adsorbed at the electrode surface [25]. Therefore, electrogenerated bromine acts as an oxidizing agent of medium strength, and also participates in reactions of electrophilic addition to multiple bonds and electrophilic substitution in aromatic systems. This behavior of the titrant makes it possible to cover a wide range of antioxidants of various nature and action mechanisms, in particular, phenolic compounds, terpenoids and their derivatives, as well as unsaturated hydrocarbons.

Electrogenerated ferricyanide ions show relatively weak oxidative properties. On the other hand, it acts as a one-electron oxidant that prevents possible accompanying steps that complicate the reaction.

#### 3.2.1. Reactivity of Essential Oils Antioxidants towards Electrogenerated Bromine

**Figure 1:**
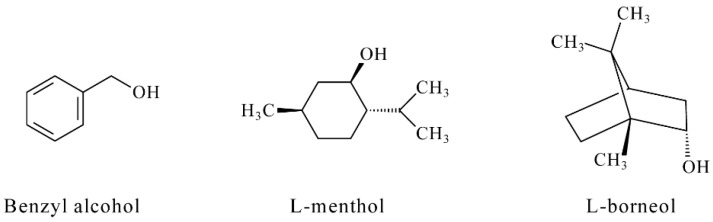
Structure of benzyl alcohol, L-menthol and L-borneol.

Reactions with electrogenerated bromine proceed rapidly and quantitatively for most of the antioxidants studied (phenolics, terpenes, and their derivatives). The number of electrons participating in the reactions calculated using Faraday’s law is consistent with the number of reactive functional groups in their structure. The only exclusions are benzyl alcohol, L-menthol and L-borneol (Figure 1) that do not interact with the titrant due to the absence of reactive functional groups in their structure.

**Figure 2:**
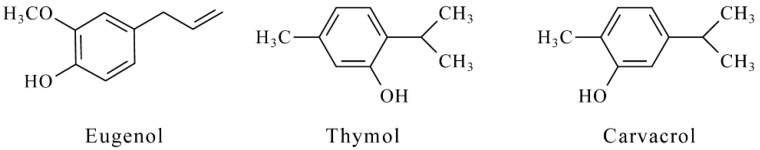
Structure of the main phenolic antioxidants of essential oils.

Eugenol, carvacrol and thymol (Figure 2) interact with electrogenerated bromine with the participation of 4, 5 and 4 electrons, respectively. Eugenol is oxidized to *o*-quinone with the participation of two electrons as well as the electrophilic addition of bromine to double bond occurs. As for the isopropylmethylphenols (carvacrol and thymol), both oxidation and bromination reactions take place, leading to the formation of several products, in particular thymoquinone and mono- and dibromo derivatives [40,41].

**Figure 3:**
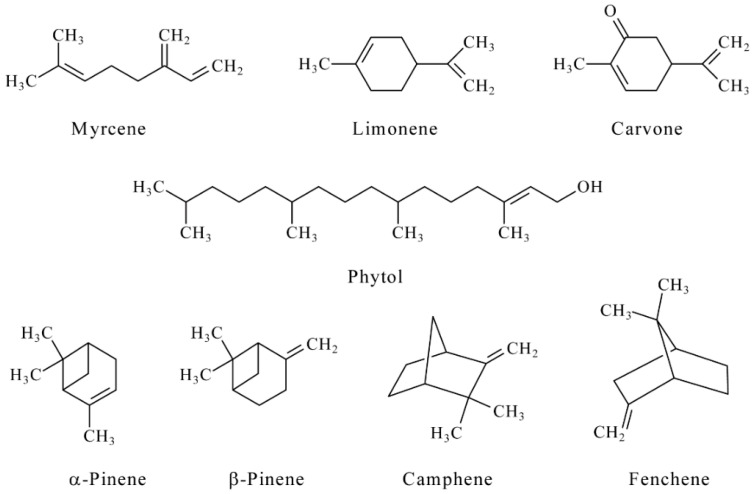
The structure of the terpenes under consideration.

Terpenes and their derivatives (Figure 3) interact with electrogenerated bromine via its electrophilic addition to double bonds.

**Scheme 1:**
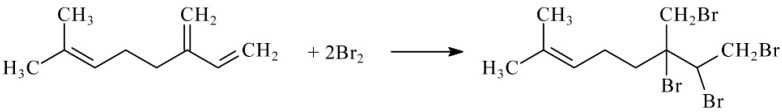
Reaction of myrcene with electrogenerated bromine.

Thus, reactions of all studied compounds except myrcene and limonene proceed with the participation of two electrons that agree well with the number of double bonds in their structure. Similar behavior has been found for limonene, for which four electrons are involved in the reaction with titrant that means bromination on both double bonds. Myrcene also reacts with electrogenerated bromine with four electrons transfer i.e., one of the double bonds is inactive, which is probably caused by sterical effects. Therefore, conjugated double bonds undergo a reaction (Scheme 1).

#### 3.2.2. Reactivity of Essential Oils Antioxidants towards Electrogenerated Ferricyianide Ions

**Scheme 2:**
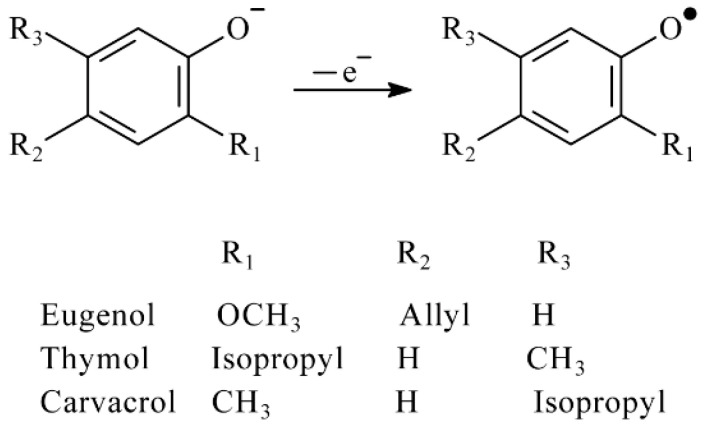
Reaction of phenolic antioxidants with electrogenerated ferricyanide ions.

Ferricyanide ions react with phenolic antioxidants, only allowing to conclude that phenolic hydroxyl groups are oxidized that agree with data for hydroxybenzoic acids [42] and flavonoids [43]. Eugenol, carvacrol and thymol undergo one electron oxidation that coincides with the number of hydroxyl groups in their structure. As long as titration with ferricyanide ions is performed in basic medium, the relatively easy electron detachment from phenolate ions takes place with the formation of the phenoxyl radical according to Scheme 2. Similar data have been reported for the reactions of these phenolic antioxidants in micellar medium of Triton X-100 [44] and Brij® 35 [45].

The number of electrons participating in the reactions of all antioxidants under consideration with electrogenerated titrants are reproduced in repeated measurements and are consistent with that reported for some of them [25], which confirms the accuracy of the data obtained. Thus, coulometric titration with electrogenerated bromine and ferricyanide ions can be applied to evaluate the antioxidant properties of essential oils.

### 3.3. Evaluation of Total Antioxidant Parameters of Essential Oils

Total antioxidant parameters of the essential oils in particular TAC and FRP based on the sample’s titration with electrogenerated bromine and ferricyanide ions, respectively, have been estimated. A screening of 15 types of essential oils has been carried out (Table 3).

The highest values of both parameters have been obtained for clove essential oils, which is caused by the high content of eugenol being the major constituent. Its concentration in clove essential oil is usually at least 50% [46]. The lower eugenol content in the essential oils of cinnamon, nutmeg and basil (2.46–0.023% according to the chromatographic determination) is reflected in their antioxidant parameters. In the case of the essential oil of thyme, a high FRP is caused by the presence of carvacrol (54.6 ± 0.1%), thymol (0.31 ± 0.05%) and trace eugenol. Isopropylmethylphenols (thymol and carvacrol) in marjoram, thymol in clary sage and trace amounts of eugenol in jasmine are the major contributors to the FRP of these essential oils. Other essential oils are almost free of phenolic antioxidants, and their components do not react with the electrogenerated ferricyanide ions. Thus, the FRP parameter reflects the contents of phenolic antioxidants in the essential oils that agree with reported for spices micellar extracts [44,45] and other plant-derived products [42,43].

High TAC (1220 ± 28 C mL^−1^) of the neroli essential oil is caused by the presence of terpenoids, in particular β-pinene, limonene, linalool and linalyl acetate reacting with electrogenerated bromine. Other essential oils that are free of volatile phenolics show significantly less TAC (83–459 C mL^−1^), which is reflected in their terpene constituents.

As can be seen in Table 3, the antioxidant parameters for the different samples of the same type of essential oils agree well with each other in the case of clove, cinnamon, lavender, and rosemary. Significantly different TAC values have been obtained for anise, bergamot, jasmine, and marjoram samples of various trademarks. This phenomenon can be caused by plant material characteristics that are strongly dependent on the origin, growing and harvesting conditions, vegetation stage, storage, and so on, as well as conditions of essential oil preparation [47]. Probably, these factors affect more strongly the terpenoid constituents and their contents as can be seen from the TAC and FRP values.

The difference in the TAC and FRP allows for concluding that TAC is a preferable parameter for screening purposes as long as it is applicable to all types of essential oils and allows for evaluating a wider range of antioxidants presented in the sample due to the different mechanisms of coulometric titrant action.

### 3.4. Correlation of Essential Oils TAC and FRP with Standard Antioxidant Parameters

Standard antioxidant parameters (antioxidant activity by reaction with DPPH^•^ and total phenolic contents) based on spectrophotometric data have been evaluated (Table 4). It should be noted that spectrophotometric determination of total phenolic content by the Folin–Ciocalteu method could not be applied to all samples under investigation due to the formation of turbid solutions after the addition of photometric reagents. Therefore, only essential oils of clove, cinnamon, nutmeg and thyme can be studied. Furthermore, the method is time-consuming (more than 1 h), requiring application of several additional reagents.

The major disadvantages of the evaluation of antioxidant activity by reaction with DPPH^•^ are the following:

- Instability of the reagent solution under light exposure;
- Necessity to use toxic methanol;
- Limited range of antioxidants reacting with the DPPH^•^;
- Certain antioxidants interact with DPPH^•^ slowly and/or reversibly that leads to distortion of results and their incorrect interpretation;
- Necessity to prepare a blank solution for each sample makes the method more tedious.

Coulometric titration does not have the limitations mentioned above, which is a favorable advantage over standard procedures. Moreover, the RSD values for coulometric determination are less than those for spectrophotometry, especially in the case of DPPH^•^-test, confirming better reproducibility of the determination.

The validation of TAC and FRP of essential oils has been carried out by comparison with antioxidant activity by reaction with DPPH^•^ and total phenolic contents. Positive significant correlations have been obtained for both parameters (Figure S1). The *r*-values (Table 5) indicate a strong correlation between the parameters according to the Chaddock scale [48].

In general, correlations obtained allow for concluding that coulometric titration with electrogenerated bromine and ferricyianide ions is a promising tool in the essential oils analysis, in particular, for the screening of their antioxidant properties.

## 4. Conclusions

Coulometric titration with electrogenerated bromine and ferricyanide ions has been suggested for the evaluation of the essential oils’ total antioxidant parameters. Data for the reaction of individual antioxidants (volatile phenolics and terpenes) with coulometric titrants confirm the applicability of the method for the characterization of essential oils TAC and FRP. Screening of 27 samples from 15 types of essential oils has been performed and statistically significant difference between the samples has been observed. The data obtained agree well with the standard spectrophotometric approaches confirming the accuracy of the coulometric methods. Disadvantages of spectrophotometric methods like instability of DPPH^•^ solution, application of several reagents, time-consuming procedures and limited applicability to essential oils samples are successfully overcome in coulometry. Moreover, the method can be easily automated, which provides high throughput (the average time of one titration is approximately 1 min) and applicability in routine analysis.

Thus, coulometric titration has been shown to be an effective and promising tool for the characterization of the essential oils via total antioxidant parameters that enlarge the practical application area of the method.

## Figures and Tables

**Table 1:** Essential oils under investigation.

Sample No.	Plant Material	Botanical Name of the Plant	Morphological Characteristics
1	Clove	*Eugenia caryophyllus* Spreng.	Leaves
2	Cinnamon	*Cinnamomum zeylanicum* Blume	Leaves
3	Nutmeg	*Myristica fragrans* Houtt	Fruits
4	Lavender	*Lavandula angustifolia* Mill.	Flowers
5	Ginger	*Zingiber officinale* Rosc.	Rhizomes
6	Anise	*Pimpinella anisum* L.	Fruits
7	Basil	*Ocimum basilicum* L.	Herb
8	Bergamot	*Citrus aurantium* subsp. *bergamia*	Fruits
9	Jasmine	*Jasminum officinale* L.	Flowers
10	Ylang-Ylang	*Cananga odorata* (Lam.) Hook. F. & Thomson	Flowers
11	Marjoram	*Origanum majorana* L.	Flowers
12	Neroli	*Citrus aurantium* subsp. *amara*	Flowers
13	Rosemary	*Rosmarinus officinalis* L.	Twigs and flowers
14	Thyme	*Thymus vulgaris* L.	Flowers
15	Clary sage	*Salvia sclarea* L.	Herb

Experiments were carried out at laboratory temperature (25 ± 2 °C).

**Table 2:** Essential oils composition according to GC-MS data (RI—retention index).

RI	Component	*m/z*	Essential Oils														
			1	2	3	4	5	6	7	8	9	10	11	12	13	14	15
885	Cyclofenchene	136															+
893	Styrene	104		+				+									
898	2,5-Diethyltetrahydrofuran	128														+	
901	Nonane	128							+								
903	2-Bornene	136											+				
909	4-Octene, 2,6-dimethyl-, [S-(E)]-	140											+				
917	5,5-Dimethyl-1-vinylbicyclo[2.1.1]hexane	136			+										+	+	
918	Tricyclene	136		+	+	+	+	+		+			+	+	+	+	+
924	α-Thujene	136			+	+		+	+	+			+	+	+	+	+
931	α-Pinene	136		+	+	+	+	+	+	+	+	+	+	+	+	+	+
935	2,2-Dimethyl-5-methylenebicyclo[2.2.1]heptane	136													+		+
945	Camphene	136		+	+	+	+	+		+	+		+	+	+	+	+
952	Thuja-2,4(10)-diene	134			+					+					+	+	
957	Benzaldehyde	106		+							+						
969	Sabinene	136					+	+	+	+			+				+
973	β-Pinene	136		+	+	+	+	+	+	+	+	+	+	+	+	+	+
978	1-Octen-3-ol	128														+	
979	*trans*-Carane	138											+				
985	3-Octanone	128				+											
987	Sulcatone	126					+		+								
996	3-Octanol	130														+	
990	Myrcene	136		+	+	+	+	+	+	+	+	+	+	+	+	+	+
1001	Pseudolimonene	136												+			
1002	α-Phellandrene	136		+	+			+		+			+		+	+	
1007	3-Carene	136					+	+		+			+	+	+	+	+
1014	α-Terpinene	136		+	+			+		+					+	+	+
1014	Hexyl acetate	144				+											
1018	*p*-Methylanisole	122										+					
1021	*p*-Menth-1-ene	138								+							
1022	*p*-Cymene	134								+							
1022	*m*-Cymene	134		+	+	+		+	+		+		+	+		+	+
1025	Limonene	136	+	+	+	+	+	+	+	+	+	+	+	+		+	
1028	Eucalyptol	154		+	+	+		+	+		+		+		+	+	+
1036	*cis*-β-Ocimene	136				+								+			+
1039	Benzyl alcohol	108									+	+					
1042	2-Propanol, 1,1′-oxybis-	134												+			
1044	Benzeneacetaldehyde	120		+													
1046	*trans*-β-Ocimene	136			+	+	+		+	+		+	+	+	+	+	
1048	1-Propanol, 2-(2-hydroxypropoxy)-	134												+			
1056	γ-Terpinene	136		+	+	+	+	+		+			+	+	+	+	+
1063	*cis*-Sabinene hydrate	154					+			+			+			+	
1071	1-Octanol	130					+			+							
1072	*cis*-Linalool oxide (furanoid)	170		+					+	+				+			
1074	*p*-Mentha-3,8-diene	136			+								+		+		+
1076	Benzyl formate	136									+						
1082	Isoterpinolene	136													+		
1084	Fenchone	152													+		
1085	Terpinolene	136		+	+	+	+	+		+			+	+		+	+
1086	*trans*-Linalool oxide (furanoid)	170							+					+			
1089	2-Nonanone	142					+										
1093	Methyl benzoate	136										+					
1094	α-Pinene oxide	152			+	+				+					+		+
1095	Benzoic acid, ME derivative	136									+						
1096	*trans*-Sabinene hydrate	154			+								+			+	
1100	Linalool	154		+	+	+	+	+	+	+	+	+	+	+	+	+	+
1103	Nonanal	142					+										
1104	*cis*-Thujone	152															+
1113	Fenchol	154			+								+				+
1113	2-Methoxy-1,7,7-trimethylbicyclo[2.2.1]heptane	168					+										
1114	1,3,8-*p*-Menthatriene	134			+												
1114	β-Thujone	152															+
1116	Phenethyl alcohol	122		+							+						
1116	4,8-Dimethyl-1,3(E),7-nonatriene	150					+										
1120	*trans*-*p*-Mentha-2,8-diene-1-ol	152								+				+			
1121	*cis*-*p*-Menth-2-en-1-ol	154			+								+				
1126	Octanoic acid, ME derivative	158					+			+							
1127	4-*trans*,6-*cis*-Alloocimene	136								+					+		
1127	α-Campholenal	152			+												
1131	*cis*-Limonene oxide	152					+			+				+			
1132	1,2-Dihydrolinalool	156		+		+						+					
1133	4-*trans*,6-*trans*-Alloocimene	152			+												
1134	*cis*-*p*-Mentha-2,8-diene-1-ol	152			+		+			+				+			
1135	*trans*-Pinocarveol	152			+								+	+			
1135	*trans*-Limonene oxide	152					+			+							
1136	Terpineol-1	154											+		+		
1137	*trans*-*p*-Menth-2-en-1-ol	154											+				
1140	*cis*-Verbenol	152								+							
1142	*trans*-Verbenol	152		+	+					+							
1144	Camphor	152				+	+				+			+	+		+
1150	*trans*-Menthone	154							+								
1151	β-Pinene oxide	152			+												
1151	Camphene hydrate	154					+										
1153	Isoborneol	154				+	+								+		+
1156	Citronellal	154					+			+							
1157	α-Pinocarvone	150			+									+			
1159	Benzenepropanal	154		+													
1159	α-Dihydroterpineol	156											+				
1162	Borneol	154				+	+			+			+		+	+	+
1165	Endo-borneol	154												+			
1164	δ-Terpineol	154											+	+			
1167	Benzyl acetate	150									+	+					
1168	Isoneral	152								+							
1170	Menthol	156							+								
1173	(−)-Terpinen-4-ol	154		+	+	+		+					+	+	+	+	+
1179	Phenylacetic acid, ME derivative	150									+						
1181	Cryptone	138					+										
1184	*p*-Cymen-8-ol	150				+							+	+			
1186	Isogeranial	152								+							
1188	α-Terpineol	154		+	+	+	+	+	+	+			+	+	+	+	+
1192	Myrtenal	150			+												
1192	Hexyl butanoate	172				+											
1194	Myrtenol	152			+	+											
1196	Estragole	148						+	+								
1196	γ-Terpineol	154		+						+			+	+			
1206	*trans*-*p*-Menth-1-en-3-ol	154											+				
1204	Verbenone	150			+	+				+					+		+
1208	Decanal	156				+	+										
1215	Isopropyl benzoate	164										+					
1216	*cis*-Cinnamaldehyde	132		+													
1216	*trans*-Carveol	152					+			+				+			
1218	Fenchyl acetate	196											+		+		+
1226	Nonanoic acid, ME	172					+										
1227	Bornyl formate	182					+										
1228	*cis*-Carveol	152					+			+				+			+
1228	*cis*-Geraniol	154				+			+	+				+			+
1232	Citronellol	156			+						+						
1236	Isogeraniol	154															+
1239	β-Citral	152			+				+	+							+
1240	Cuminaldehyde	148		+			+										
1240	Carvone	150				+	+							+	+		
1241	Carvacrol, ME derivative	164														+	
1250	*cis*-Anethole	148						+									
1256	Linalyl acetate	196		+		+				+	+	+	+	+			+
1257	Geraniol	154		+	+		+		+								
1269	α-Citral	152			+		+		+	+				+			+
1271	*trans*-Ascaridol glycol	170													+		
1273	*trans*-*p*-Menth-2-ene-1,4-diol	170											+				
1276	*trans*-Cinnamaldehyde	132		+													
1278	β-Terpinyl acetate	196			+					+							
1282	Bornyl acetate	196			+	+	+			+			+	+	+		+
1283	*trans*-Anethole	148						+	+				+				
1284	Safrole	162			+												
1290	Indole	117									+						
1290	*trans*-Sabinyl acetate	194															+
1290	Lavandulyl acetate	196				+											
1291	Thymol	150											+			+	+
1293	Menthyl acetate	198							+								
1294	2-Undecanone	170					+										
1299	2-Cyclohexen-1-ol, 3-methyl-6-(1-methylethyl)-, acetate	196											+				
1300	Carvacrol	150											+			+	
1305	1-Methoxy-4-pentylbenzene	178			+												
1311	δ-Terpinyl acetate	196								+							+
1313	2-Propen-1-ol, 3-phenyl-, (E)-	134		+							+						
1320	Chavicol, TMS derivative	134	+														
1324	Myrtenyl acetate	194															+
1325	Decanoic acid, ME derivative	186					+			+							
1329	(−)-*trans*-*p*-Menth-8-en-2-ol, acetate	196								+							
1335	2-Propen-1-ol, 3-phenyl-, formate	162		+													
1335	δ-EIemene	204					+						+				
1337	Linalyl propionate	210				+								+			
1344	Anthranilic acid, ME derivative	151												+			
1346	α-Terpinyl acetate	196			+					+			+	+			+
1347	α-Cubebene	204	+			+	+		+			+			+		
1350	*p*-Acetylanisole	150							+								
1354	Citronellol acetate	198					+										
1357	Eugenol	164	+	+	+				+		+					+	
1358	Triacetin	218								+							
1363	Cyclosativene	204					+										
1364	Neryl acetate	196				+			+	+		+	+	+			+
1370	Carvacrol acetate	192														+	
1370	α-Copaene	204		+	+	+	+	+	+			+	+	+	+		+
1380	*p*-Acetonylanisole	164						+									
1380	Daucene	204				+											
1381	β-Bourbonene	204				+			+						+		+
1382	Geranyl acetate	196			+	+				+	+	+	+	+			+
1385	Cinnamic acid, ME	162		+													
1387	epi-Sesquithujene	204				+											
1390	β-Elemene	204			+		+	+	+	+		+	+	+	+		+
1400	Longifolene	204				+			+				+	+			+
1401	Methyleugenol	178			+				+								
1401	Isocaryophyllene	204											+		+		
1402	Sesquithujene	204					+										
1403	*cis*-β-Caryophyllene	204										+		+			+
1406	α-Gurjunene	204								+							
1407	Decyl acetate	200				+											
1407	α-Cedrene	204															+
1411	*cis*-α-Bergamotene	204						+									
1413	*trans*-β-Caryophyllene	204	+	+	+	+	+	+	+	+	+	+	+	+	+	+	+
1427	*cis*-Thujopsene	204								+							+
1428	β -Copaene	204			+	+	+		+			+		+			
1429	γ-Elemene	204			+		+										
1431	*trans*-α-Bergamotene	204			+	+	+	+	+	+							
1435	10,10-Dimethyl-2,6-dimethylenebicyclo[7.2.0]undecane	204		+									+				
1436	Aromandendrene	204				+		+					+	+		+	
1437	α-Guaiene	204	+												+		
1438	α-Maaliene	204											+				
1438	β-Humulene	204														+	
1440	*cis*-β-Farnesene	204							+								
1443	Epi-β-Santalene	204							+								
1445	Coumarin	146				+											
1446	Cinnamyl acetate	176		+													
1449	α-Humulene	204	+	+	+	+		+	+			+	+	+	+	+	
1455	Alloaromadendrene	204						+					+		+	+	+
1455	*trans*-β-Farnesene	204			+	+		+	+					+			
1473	γ-Muurolene	204	+	+	+			+	+			+	+	+		+	+
1475	Bicyclo[4.3.0]nonane, 7-methylene-2,4,4-trimethyl-2-vinyl-	204	+														
1476	4-epi-α-Acoradiene	204											+				
1477	Germacrene D	204			+	+	+		+			+					
1480	α-Curcumene	202					+						+				
1486	Naphthalene, 1,2,4*a*,5,6,8*a*-hexahydro-4,7-dimethyl-1-(1-methylethyl)-	204	+														
1487	β-Selinene	204	+		+		+										+
1488	Valencene	204								+				+			
1489	α-Selinene	204	+						+								
1490	Bicyclogermacrene	204						+					+				
1492	Leden	204						+						+		+	+
1496	Epizonarene	204	+														
1496	α-Muurolene	204	+	+	+			+	+			+	+	+		+	+
1499	Zingiberene	204					+										
1503	Cuparene	202															+
1503	*cis*-α-Bisabolene	204							+								
1508	*trans*-α-Farnesene	204	+									+	+				
1507	β-Bisabolene	204		+	+	+	+	+	+	+						+	
1511	γ-Cadinene	204	+	+	+	+		+					+			+	+
1515	2,4-Di-tert-butylphenol	206											+		+		
1515	Bornyl 3-methylbutanoate	238												+			
1519	Myristicine	192			+												
1518	β-Cadinene	204						+	+					+		+	
1520	δ-Cadinene	204	+	+						+		+	+				+
1521	β-Sesquiphellandrene	204				+	+										
1523	Lilial	204									+						
1523	Dodecanoic acid, ME derivative	214								+							
1524	Lauric acid, ME derivative	214												+			
1528	γ-Bisabolene	204					+										
1528	Cubenene	204	+					+									+
1528	Eugenol acetate	206		+													
1529	*cis*-psi-Ionone	192							+	+							
1533	*cis*-Nerolidol	222								+				+			
1535	α-Cadinene	204	+		+			+									
1537	Selina-3,7(11)-diene	204											+				
1544	α-Calacorene	200	+														
1545	Elemol	222					+										
1550	Germacrene B	204			+		+										
1554	Diepicedrene-1-oxide	220	+														
1556	Elemicin	208			+												
1560	Methyl N-formylanthranilate, ME derivative	179												+			
1561	*trans*-Nerolidol	222					+	+	+	+				+			
1563	Palustrol	222															+
1564	Ledol	222											+				
1566	7-Methyl-1-naphthol	158		+													
1566	*p*-Menth-6-en-2-one, 8-hydroxy-, acetate	210								+							
1570	Caryophyllene alcohol	222	+														
1574	Spathulenol	220			+			+	+				+			+	
1578	Globulol	222						+									
1577.5	Caryophyllene oxide I	220	+	+	+	+			+	+		+	+	+		+	+
1581.5	psi-Ionone	192							+	+							
1587	*trans*-Sesquisabinene hydrate	222					+										
1587	Viridiflorol	222						+					+			+	+
1594	α-Cedrol	222								+							+
1595	Rosifoliol	222						+								+	
1602	Methoxyeugenol	194			+												
1602	Humulene-1,2-epoxide	220													+		
1605	Humulene oxide II	220	+	+	+				+			+		+		+	+
1611	Zingiberenol	222					+										
1618	Isopropyl laurate	242										+					
1624	1-epi-Cubenol	222		+				+									
1627	γ-Eudesmol	222						+									
1636	Isospathulenol	220											+				
1636	Caryophylla-4(12),8(13)-dien-5α-ol	220	+	+									+	+	+	+	
1637	α-epi-Muurolol	222		+	+				+			+					
1637	α-epi-Cadinol	222				+		+								+	+
1640	10,10-Dimethyl-2,6-dimethylenebicyclo[7.2.0]undecan-5β-ol	220														+	
1643	11,11-Dimethyl-4,8-dimethylenebicyclo[7.2.0]undecan-3-ol	220	+	+													
1643	δ-Cadinol	222						+									
1646	β-Eudesmol	222					+	+	+				+			+	+
1650	α-Cadinol	222			+			+				+				+	+
1654	α-Eudesmol	222											+			+	
1674	Aromadendrene oxide-(2)	220	+													+	+
1681	1-(3-Methyl-2-butenoxy)-4-(1-propenyl)benzene	202						+									
1683	Heptadecene	238	+														
1684	α-Bisabolol	222		+		+			+	+							
1693	*trans*-Stilbene	180		+													
1713	2-*trans*,6-*cis*-Farnesol	222						+									
1726	Myristic acid, ME derivative	242										+		+			
1746	α-Hexylcinnamaldehyde	216									+		+	+			
1766	Benzyl benzoate	212		+	+							+	+				
1791	Ethyl myristate	256										+					
1820	Phenylacetic acid, benzyl ester	226									+						
1828	Isopropyl myristate	270	+	+				+			+	+		+			
1841	Phenylacetic acid, 2-methylphenyl ester	226									+						
1844	Hexahydrofarnesyl acetone	268						+									
1845	6,10,14-Trimethyl-2-pentadecanone	268														+	
1852	Benzoic acid, 2-phenylethyl ester	226									+						
1859	Myristic acid, TMS derivative	300			+										+		
1895	Propyl myristate	270										+					
1902	Isopimara-9(11),15-diene	272															+
1903	Nonadecane	268									+						
1913	Phenylacetic acid, phenethyl ester	240									+						
1928	Palmitic acid, ME derivative	270	+			+	+	+		+		+		+			
1953	*m*-Camphorene	272			+								+	+		+	+
1966	Geranyl benzoate	258			+												
1971	Palmitic acid	256									+						
1984	*p*-Camphorene	272			+								+	+			+
1995	Palmitic acid, ethyl ester	284									+						
2025	Isopropyl palmitate	298										+					
2045	Palmitic acid, TMS derivative	328													+		
2048	Dehydroabietane	270														+	
2051	Epimanool	290															+
2066	13-Hexyloxacyclotridec-10-en-2-one	280									+						
2078	Abieta-7,13-diene	272														+	
2081	Isoeugenol, benzyl ether	254									+						
2093	Linoleic acid, ME derivative	294					+	+		+	+	+		+			
2094	*trans*-Cinnamyl benzoate	238		+													
2098	13-Octadecenoic acid, ME derivative	296						+			+			+			
2103	Heneicosane	296									+						
2105	Elaidic acid, ME derivative	296								+							
2107	10-Octadecenoic acid, ME derivative	296									+						
2108	6-Methyl-4,6-bis(4-methylpent-3-en-1-yl)cyclohexa-1,3-dienecarbaldehyde	286							+								
2108	*trans*-Vaccenic acid, ME derivative	296								+							
2111	Phytol	296							+								
2128	Stearic acid, ME derivative	298					+			+		+					
2167	Linoleic acid, ethyl ester	308									+						
2177	Ethyl Oleate	310									+						
2196	Isopropyl 9-octadecenoate	324										+					
2301	11-Eicosenoic acid, ME derivative	324									+						
2329	Eicosanoic acid, ME derivative	326								+	+						
2370	9-Octadecenoic acid, 12-hydroxy-, ethyl ester, [R-(Z)]-	326									+						
2442	Cinnamyl cinnamate	264		+													
2528	Behenic acid, ME derivative	354								+	+						
2729	Lignoceric acid, ME derivative	382									+						
3494	Decanoic acid, 1,2,3-propanetriyl ester	554								+							

**Table 3:** Total antioxidant parameters of the essential oils based on the coulometric titration data (*n* = 5; *p* = 0.95).

Essential Oil	Sample *	TAC (C mL^−1^)	RSD (%)	FRP (C mL^−1^)	RSD (%)
Clove	1a	2600 ± 100	4.2	11.5 ± 0.6	3.3
	1b	2400 ± 100	3.4	20.6 ± 0.8	2.9
	1c	2570 ± 40	1.4	9.5 ± 0.5	4.6
Cinnamon	2a	740 ± 30	3.5	3.2 ± 0.1	3.0
	2b	770 ± 30	3.0	3.2 ± 0.1	3.0
	2c	660 ± 30	4.1	3.3 ± 0.1	2.9
Nutmeg	3	320 ± 20	4.1	3.9 ± 0.3	2.9
Lavender	4a	143 ± 3	1.5	—	
	4b	104 ± 2	0.77	—	
Ginger	5	190 ± 9	4.7	—	
Anise	6a	127 ± 6	2.0	—	
	6b	459 ± 20	3.5	—	
Basil	7a	145 ± 6	1.7	1.50 ± 0.06	3.9
Bergamot	8a	200 ± 10	4.6	—	
	8b	123 ± 4	2.5	—	
	8c	230 ± 7	2.6	—	
Jasmine	9a	191 ± 8	3.2	1.6 ± 0.1	3.7
	9b	510 ± 13	1.6	1.45 ± 0.08	4.2
	9c	174 ± 3	1.1	2.4 ± 0.1	2.1
Ylang-Ylang	10	220 ± 9	3.3	—	
Marjoram	11a	139 ± 7	4.2	3.0 ± 0.2	4.7
	11b	515 ± 8	0.59	2.4 ± 0.1	3.9
Neroli	12	1220 ± 28	1.8	—	
Rosemary	13a	93 ± 5	4.6	—	
	13b	83 ± 3	3.3	—	
Thyme	14	1540 ± 20	0.97	4.8 ± 0.2	3.7
Clary sage	15	71 ± 2	1.4	1.7 ± 0.1	5.6

* the letters correspond to various trademarks of essential oils.

**Table 4:** Standard antioxidant parameters of essential oils based on spectrophotometry data (*n* = 3; *p* = 0.95).

Essential Oil	Sample *	Antioxidant Activity (%)	RSD (%)	Total Phenolic Contents (mg Eugenol mL^−1^)	RSD (%)
Clove	1a	94.4 ± 0.7	0.30	738 ± 43	2.4
	1b	94.6 ± 0.6	0.45	789 ± 6	0.32
	1c	94.8 ± 0.2	0.079	694 ± 42	2.5
Cinnamon	2a	60.4 ± 0.5	0.36	41 ± 2	2.4
	2b	63 ± 2	1.6	39 ± 2	2.6
	2c	61.8 ± 0.4	0.24	45 ± 5	4.4
Nutmeg	3	11.3 ± 0.5	1.9	4.3 ± 0.1	2.0
Lavender	4a	2.3 ± 0.1	3.9	—	
	4b	1.80 ± 0.08	4.1	—	
Ginger	5	2.4 ± 0.4	6.0	—	
Anise	6a	1.4 ± 0.1	6.9	—	
	6b	40 ± 2	4.7	—	
Basil	7a	3.5 ± 0.2	5.5	—	
Bergamot	8a	2.2 ± 0.1	4.3	—	
	8b	2.1 ± 0.1	4.4	—	
	8c	2.7 ± 0.2	6.8	—	
Jasmine	9a	4.5 ± 0.3	6.2	—	
	9b	58 ± 1	1.5	—	
	9c	12.6 ± 0.7	5.2	—	
Ylang-Ylang	10	2.5 ± 0.1	3.8	—	
Marjoram	11a	2.6 ± 0.1	3.5	—	
	11b	40.8 ± 0.2	0.45	—	
Neroli	12	0.81 ± 0.04	4.8	—	
Rosemary	13a	1.9 ± 0.1	5.1	—	
	13b	0.50 ± 0.03	5.8	—	
Thyme	14	37 ± 3	2.8	125 ± 12	4.0
Clary sage	15	1.45 ± 0.09	6.0	—	

* the letters correspond to various trademarks of essential oils.

**Table 5:** Correlation coefficients of the antioxidant parameters of essential oils.

Antioxidant Parameter Based on Coulometry	Antioxidant Activity towards DPPH^•^ (%)		Total Phenolic Contents (mg Eugenol mL^−1^)	
	r	r_critical_	r	r_critical_
TAC (C mL^−1^)	0.8379	0.3809	0.9558	0.7067
FRP (C mL^−1^)	0.7051	0.5140	0.8886	

## Data Availability

Data is contained within the article and Supplementary Materials.

## Supplementary Materials

The following supporting information can be downloaded at: https://www.mdpi.com/article/10.3390/antiox11091749/s1, Figure S1: Correlation plots of the essential oils antioxidant parameters. (**a**) TAC vs. DPPH^•^; (**b**) FRP vs. DPPH^•^; (**c**) TAC vs. total phenolics; (**d**) FRP vs. total phenolics.

## References

[1] Zuzarte M., Salgueiro L., de Sousa D.P. (2015). Essential oils chemistry. Bioactive Essential Oils and Cancer.

[2] Esposito E.R., Bystrek M.V., Klein J.S. (2014). An elective course in aromatherapy science. Am. J. Pharm. Educ..

[3] Ali B., Al-Wabel N.A., Shams S., Ahamad A., Khan S.A., Anwar F. (2015). Essential oils used in aromatherapy: A systemic review. Asian Pac. J. Trop. Biomed..

[4] Fernández-López J., Viuda-Martos M. (2018). Introduction to the special issue: Application of essential oils in food systems. Foods.

[5] Basavegowda N., Baek K.-H. (2021). Synergistic antioxidant and antibacterial advantages of essential oils for food packaging applications. Biomolecules.

[6] Schiller C., Schiller D. (1994). 500 Formulas for Aromatherapy: Mixing Essential Oils for Every Use.

[7] Wildwood C. (1996). The Encyclopedia of Aromatherapy.

[8] Amorati R., Foti M.C., Valgimigli L. (2013). Antioxidant activity of essential oils. J. Agric. Food Chem..

[9] Anthony K.P., Deolu-Sobogun S.A., Saleh M.A. (2012). Comprehensive assessment of antioxidant activity of essential oils. Food Sci..

[10] Olmedo R., Ribotta P., Grosso N.R. (2018). Antioxidant activity of essential oils extracted from *Aloysia triphylla* and *Minthostachys mollis* that improve the oxidative stability of sunflower oil under accelerated storage conditions. Eur. J. Lipid Sci. Technol..

[11] Sengun I.Y., Yucel E., Ozturk B., Kilic G. (2021). Chemical compositions, total phenolic contents, antimicrobial and antioxidant activities of the extract and essential oil of *Thymbra spicata* L. growing wild in Turkey. Food Meas..

[12] Torres-Martínez R., García-Rodríguez Y.M., Ríos-Chávez P., Saavedra-Molina A., López-Meza J.E., Ochoa-Zarzosa A., Garciglia R.S. (2017). Antioxidant activity of the essential oil and its major terpenes of *Satureja macrostema* (Moc. and Sessé ex Benth.) Briq. Pharmacogn. Mag..

[13] Hashemi S.M.B., Khorram S.B., Sohrabi M., Hashemi S.M.B., Mousavi Khaneghah A., de Souza Sant’Ana A. (2017). Antioxidant activity of essential oils in foods in essential oils in food processing. Essential Oils in Food Processing: Chemistry, Safety and Applications.

[14] Crespo Y.A., Sánchez L.R.B., Quintana Y.G., Cabrera A.S.T., del Sol A.B., Mayancha D.M.G. (2019). Evaluation of the synergistic effects of antioxidant activity on mixtures of the essential oil from *Apium graveolens* L., *Thymus vulgaris* L. and *Coriandrum sativum* L. using simplex-lattice design. Helyon.

[15] Boveiri Dehsheikh A., Mahmoodi Sourestani M., Boveiri Dehsheikh P., Vitalini S., Iriti M., Mottaghipisheh J. (2019). A Comparative Study of Essential Oil Constituents and Phenolic Compounds of Arabian Lilac (*Vitex trifolia* var. Purpurea): An Evidence of Season Effects. Foods.

[16] Semiz G., Semiz A., Mercan-Doğan N. (2018). Essential oil composition, total phenolic content, antioxidant and antibiofilm activities of four *Origanum* species from southeastern Turkey. Int. J. Food Prop..

[17] Ziyatdinova G., Kozlova E., Morozova E., Budnikov H. (2018). Chronocoulometric method for the evaluation of antioxidant capacity of medicinal plant tinctures. Anal. Methods.

[18] Ziyatdinova G., Snegureva Y., Budnikov H. (2017). Novel approach for the voltammetric evaluation of antioxidant activity using DPPH^•^-modified electrode. Electrochim. Acta.

[19] Ziyatdinova G., Zelenova Y., Budnikov H. (2020). Novel modified electrode with immobilized galvinoxyl radical for the voltammetric determination of antioxidant activity. J. Electroanal. Chem..

[20] Abdullin I.F., Turova E.N., Gaisina G.K., Budnikov G.K. (2002). Use of electrogenerated bromine for estimating the total antioxidant capacity of plant raw materials and plant-based medicinal preparations. J. Anal. Chem..

[21] Abdullin I.F., Turova E.N., Budnikov G.K., Ziyatdinova G.K., Gajsina G.K. (2002). Electrogenerated bromine-reagent for determination of antioxidant capacity of juices and extracts. Zavod. Lab. Diagn. Mater..

[22] Ziyatdinova G.K., Zakharova S.P., Budnikov H.C. (2015). Reactions of phenolic antioxidants with electrogenerated superoxide anion radical and their analytical application. Uch. Zap. Kazan. Univ. Ser. Estestv. Nauki.

[23] Ghiaba Z., Yousfi M., Hadjadj M., Saidi M., Dakmouche M. (2014). Study of antioxidant properties of five algerian date (*Phoenix dactylifera* L.) cultivars by cyclic voltammetric technique. Int. J. Electrochem. Sci..

[24] Gonçalves R.S., Battistin A., Pauletti G., Rota L., Serafini L.A. (2009). Antioxidant properties of essential oils from *Mentha* species evidenced by electrochemical methods. Rev. Bras. Plantas Med..

[25] Ziyatdinova G., Budnikov H. (2021). Analytical capabilities of coulometric sensor systems in the antioxidants analysis. Chemosensors.

[26] Abdullin I.F., Budnikov G.K. (1998). Coulometric analysis of organic compounds (review). Ind. Lab..

[27] Abdullin I.F., Budnikov G.K., Gorbunova T.S. (1997). Electrochemical generation of hypohalogenite ions and their application to determining pharmaceuticals. J. Anal. Chem..

[28] Howes M.-J.R., Simmonds M.S.J., Kite G.C. (2004). Evaluation of the quality of sandalwood essential oils by gas chromatography–mass spectrometry. J. Chromatogr. A.

[29] Kupriyanova O.V., Shevyrin V.A., Shafran Y.M., Lebedev A.T., Milyukov V.A., Rusinov V.L. (2020). Synthesis and determination of analytical characteristics and differentiation of positional isomers in the series of *N*-(2-methoxybenzyl)-2-(dimethoxyphenyl)ethanamine using chromatography–mass spectrometry. Drug Test. Anal..

[30] (2013). Vegetable Oils and Animal Fats. Preparation of Methyl Esters of Fatty Acids.

[31] Toivo J., Piironen V., Kalo P., Varo P. (1998). Gas chromatographic determination of major sterols in edible oils and fats using solid-phase extraction in sample preparation. Chromatographia.

[32] Babushok V.I., Linstrom P.J., Zenkevich I.G. (2011). Retention indices for frequently reported compounds of plant essential oils. J. Phys. Chem. Ref. Data.

[33] Tkachev A.V. (2008). Issledovanie Letuchikh Veshchestv Rastenii.

[34] NIST: National Institute of Standards and Technologies, Mass Spectra Libraries. http://www.sisweb.com/software/nist-gc-library.htm.

[35] Brand-Williams W., Cuvelier M.E., Berset C. (1995). Use of a free radical method to evaluate antioxidant activity. LWT—Food Sci. Technol..

[36] Singleton V.L., Rossi J.A. (1965). Colorimetry of total phenolics with phosphomolybdic-phosphotungstic acid reagents. Am. J. Enol. Vitic..

[37] Parthasarathy V.A., Chempakam B., Zachariah T.J. (2008). Chemistry of Spices.

[38] Hüsnü K., Başer K.H.C., Demirci F., Berger R.G. (2007). Chemistry of essential oils. Flavours and Fragrances.

[39] Adams R.P. (2007). Identification of Essential Oil Components by Gas Chromatography/Mass Spectrometry.

[40] Sabuzi F., Churakova E., Galloni P., Wever R., Hollmann F., Floris B., Conte V. (2015). Thymol bromination—A comparison between enzymatic and chemical catalysis. Eur. J. Inorg. Chem..

[41] Günay T., Çimen Y., Karabacak R.B., Türk H. (2016). Oxidation of thymol and carvacrol to thymoquinone with KHSO_5_ catalyzed by iron phthalocyanine tetrasulfonate in a methanol–water mixture. Catal. Lett..

[42] Ziyatdinova G., Salikhova I., Budnikov H. (2014). Coulometric titration with electrogenerated oxidants as a tool for evaluation of cognac and brandy antioxidant properties. Food Chem..

[43] Ziyatdinova G., Nizamova A., Budnikov H. (2011). Novel coulometric approach to evaluation of total free polyphenols in tea and coffee beverages in presence of milk proteins. Food Anal. Methods.

[44] Ziyatdinova G.K., Cong F.N., Budnikov H.C. (2015). Assessment of the antioxidant properties of micellar spice extracts by galvanostatic coulometry with electrogenerated hexacyanoferrate(III) ions. J. Anal. Chem..

[45] Ziyatdinova G., Ziganshina E., Cong P.N., Budnikov H. (2016). Ultrasound-assisted micellar extraction of phenolic antioxidants from spices and antioxidant properties of the extracts based on coulometric titration data. Anal. Meth..

[46] Haro-González J.N., Castillo-Herrera G.A., Martínez-Velázquez M., Espinosa-Andrews H. (2021). Clove essential oil (*Syzygium aromaticum* L. Myrtaceae): Extraction, chemical composition, food applications, and essential bioactivity for human health. Molecules.

[47] Barra A. (2009). Factors affecting chemical variability of essential oils: A review of recent developments. Nat. Prod. Commun..

[48] Archdeacon T.J. (1994). Correlation and Regression Analysis: A Historian’s Guide.

