# Supplementary material for: Constant–Current Coulometry with Electrogenerated Titrants as a Novel Tool for the Essential Oils Screening Using Total Antioxidant Parameters
Source: Antioxidants (Basel). 2022 Sep 3;11(9):1749. doi: 10.3390/antiox11091749 (PMC9495329; doi:10.3390/antiox11091749)
Supplement: Supplementary file 1 [file antioxidants-11-01749-s001.zip › antioxidants-1895732-supplementary.pdf]

Electronic supplementary data

Constant-current coulometry with electrogenerated titrants as a novel tool for the essential oils screening using total antioxidant parameters

Alena Kalmykova, Guzel Ziyatdinova and Olga Kupriyanova

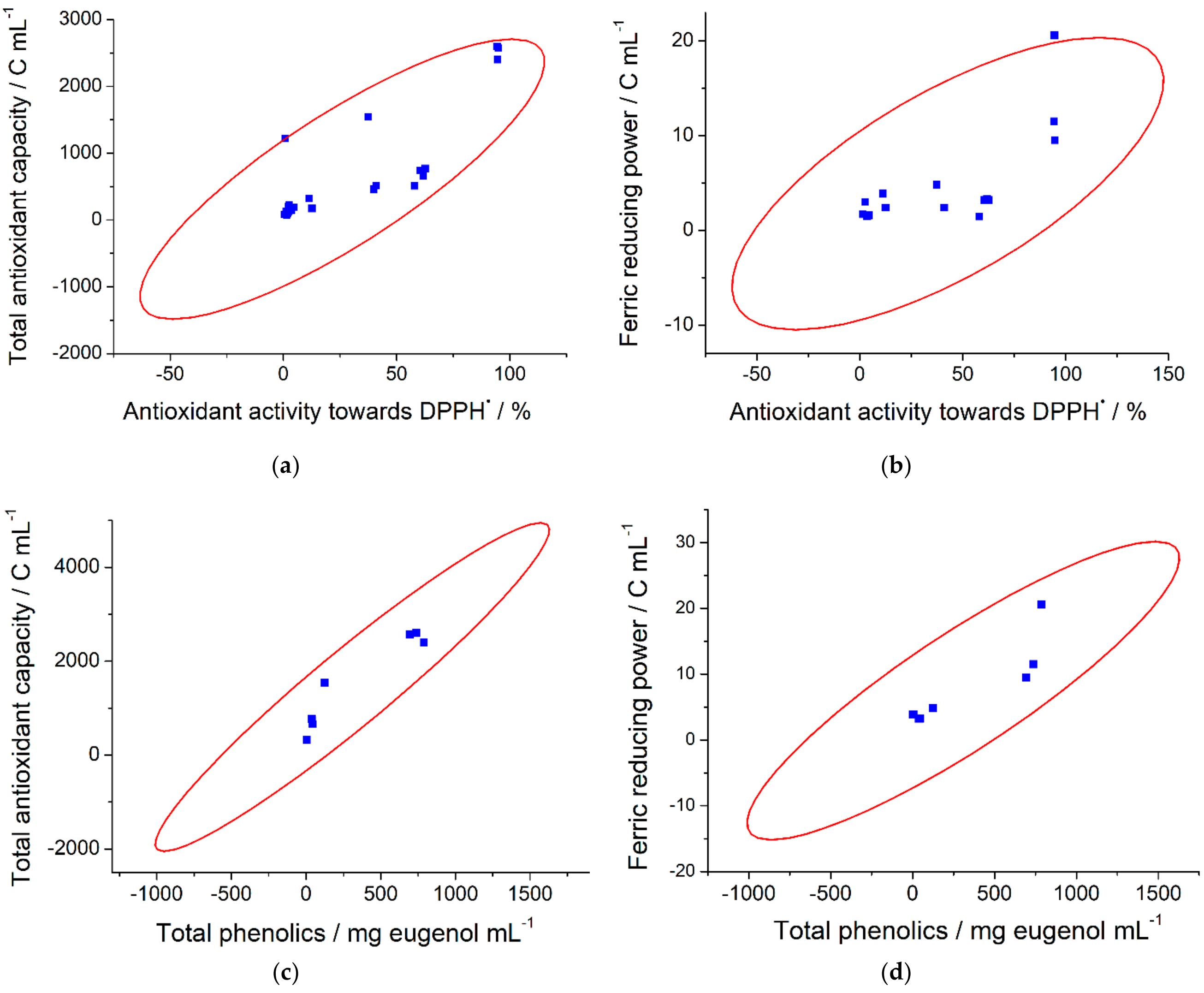

Figure S1. Correlation plots of the essential oils antioxidant parameters. (**a**) TAC *vs*. DPPH•; (**b**) FRP *vs*. DPPH•; (**c**) TAC *vs*. total phenolics; (**b**) FRP *vs*. total phenolics.
